# Growth, Yield and Fruit Quality of Grapevines under Organic and Biodynamic Management

**DOI:** 10.1371/journal.pone.0138445

**Published:** 2015-10-08

**Authors:** Johanna Döring, Matthias Frisch, Susanne Tittmann, Manfred Stoll, Randolf Kauer

**Affiliations:** 1 Department of General and Organic Viticulture, Hochschule Geisenheim University, Geisenheim, Germany; 2 Institute of Agronomy and Plant Breeding II, Justus Liebig University, Gießen, Germany; Fresno, UNITED STATES

## Abstract

The main objective of this study was to determine growth, yield and fruit quality of grapevines under organic and biodynamic management in relation to integrated viticultural practices. Furthermore, the mechanisms for the observed changes in growth, yield and fruit quality were investigated by determining nutrient status, physiological performance of the plants and disease incidence on bunches in three consecutive growing seasons. A field trial (*Vitis vinifera* L. cv. Riesling) was set up at Hochschule Geisenheim University, Germany. The integrated treatment was managed according to the *code of good practice*. Organic and biodynamic plots were managed according to Regulation (EC) No 834/2007 and Regulation (EC) No 889/2008 and according to ECOVIN- and Demeter-Standards, respectively. The growth and yield of the grapevines differed strongly among the different management systems, whereas fruit quality was not affected by the management system. The organic and the biodynamic treatments showed significantly lower growth and yield in comparison to the integrated treatment. The physiological performance was significantly lower in the organic and the biodynamic systems, which may account for differences in growth and cluster weight and might therefore induce lower yields of the respective treatments. Soil management and fertilization strategy could be responsible factors for these changes. Yields of the organic and the biodynamic treatments partially decreased due to higher disease incidence of downy mildew. The organic and the biodynamic plant protection strategies that exclude the use of synthetic fungicides are likely to induce higher disease incidence and might partially account for differences in the nutrient status of vines under organic and biodynamic management. Use of the biodynamic preparations had little influence on vine growth and yield. Due to the investigation of important parameters that induce changes especially in growth and yield of grapevines under organic and biodynamic management the study can potentially provide guidance for defining more effective farming systems.

## Introduction

The negative impact of agriculture on the environment has increased since agricultural production intensified [1,2]. Organic farming systems with their holistic approach can be seen as a possibility to face these problems and to minimize the negative impact of agriculture on the environment [3]. “Organic agriculture is a holistic production management system which promotes and enhances agro-ecosystem health, including biodiversity, biological cycles, and soil biological activity. It emphasizes the use of management practices in preference to the use of off-farm inputs […]. This is accomplished by using, where possible, agronomic, biological, and mechanical methods, as opposed to using synthetic materials, to fulfill any specific function within the system” [4]. Organic agricultural practice has to be adapted to local farming, climatic, geographical as well as social factors [3]. Research on organic farming can help adapt the system to these local factors and can furthermore investigate the effects of the production system on the ecosystem, soil, plants, food quality and economic performance under different conditions and therefore help to improve the production systems.

Demand and production of organic crops have been growing exponentially in the last few decades around the world [5,6]. Perennial crops account for about 3.2 million hectares of agricultural land worldwide. With almost nine percent, perennial cropland has a higher share in organic agriculture compared to total agriculture. Together with coffee and olives, grapes are among the most important perennial crops [7]. In most winegrowing countries organic viticulture is gaining more and more importance, but in most non-European countries organic viticulture is still in the initial stages [8]. The organically managed viticultural area in Europe increased substantially from 43000 ha in 1998 to 230000 ha in 2011, corresponding to around 5.3% of all vineyards [7,9]. Worldwide, 2.3% of all vineyards are managed according to organic standards. Furthermore, some of the world`s most prestigious wine producers have converted to organic and biodynamic viticulture [10]. This might be one reason for the increased interest in these management systems from both consumers and producers.

### Research on organic versus conventional farming

Comparisons of organic and conventional farming have long been a common topic and a great deal of knowledge on organic agriculture has been accumulated. Many studies concentrated on soil quality, yield, economic performance and environmental impact, among them several long-term field trials. Organically farmed soil had significantly higher soil organic matter content [11–13], less soil erosion, larger topsoil depth [11], showed increased biological activity [13–15], lower bulk density [14–16], and higher soil quality [17,18] for various crops. The organic system showed higher soil nitrogen content [13] and reduced carbon and nitrogen losses [12], but showed lower phosphorus levels compared to conventional treatments under Australian conditions [19]. Yield under organic management decreased from 14 up to 67% compared to conventional agriculture for many crops such as potatoes, winter wheat, grass-clover [20], grain, sunflower, common wheat, sugar beet [21], cotton [22], soybean [23] and maize during conversion [13]. Other studies did not detect significant differences in soybean yields [22], maize yields [12,13] and pigeon bean yields [21] between organic and conventional production. Organically grown pears, peaches and apples did not differ in yield from conventionally produced fruit [16,17,24]. Concerning the environmental impact, the organic systems showed efficient resource utilization as well as enhanced floral and faunal diversity [20] and maintained soil productivity [11,12]. Organic cropping systems are therefore considered more sustainable than conventional cropping systems from an environmental standpoint.

Lately, a lot of research has been done on food quality of vegetables and fruit, among them several perennial crops. Organically grown tomatoes had smaller fruit size and mass, but were of better quality, had higher soluble solids, higher vitamin C content [25] and a significantly higher amount of flavonoids compared to conventionally produced tomatoes [26]. This might be due to increased oxidative stress during fruit development [25]. Organically produced strawberries were of higher quality [18]. Organically produced pears did not vary significantly in storage life, fruit weight, pH and soluble solids from conventional pears [24], but organically produced apples were sweeter and less tart than conventional apples [17].

### Research on the biodynamic farming system

Investigations on biodynamic farming systems are scarce in contrast to organic farming systems, which attracted considerable interest in the scientific community. The biodynamic agricultural movement started in the 1920s and it has been further developed in the following decades and has been institutionalized by the international certification label DEMETER. Biodynamic farming can be regarded as a form of organic agriculture. In addition to methods in organic cropping, biodynamic farming emphasizes biodiversity, influence of celestial bodies and the concept of the farm as an organism. Furthermore, a series of fermented manure, plant, and mineral preparations (divided into field spray and compost preparations) are applied on soil, crops, and compost [27]. These preparations are claimed to stimulate soil nutrient cycling and compost development and to promote photosynthesis. While the biodynamic farming system is recognized as an organic cropping system and its advantages as such are undoubted, the effects of the biodynamic preparations are still unconfirmed.

Research on biodynamic farming revealed a behavior similar to the organic farming system concerning soil characteristics, yield and growth of agricultural crops and economic performance, resource utilization and biodiversity [19,20,22,28–32]. Some authors report an increase of storage life of crops under biodynamic production [29,32] or minor differences in product quality [19,20,32], but results are not consistent. That is why it is still controversial whether biodynamic preparations as such have any effects or benefits [28–30,32–48].

### Research on organic and biodynamic viticulture

In viticulture, few studies exist concerning the influence of organic management on growth, yield and grape or wine quality. The number of scientific studies investigating biodynamic viticulture is even more restricted. The major effects of organic compared to integrated or conventional viticulture are increased soil microbiological activity [49,50], increased soil organic carbon [49,51], decreased growth expressed as reduced pruning weight and reduced shoot length [52–55] as well as decreased yields [52–57]. In some cases reduced berry weight [55,57,58] and reduced number of berries per cluster [53], increased disease frequency of *Botrytis cinerea* (Botrytis) [56] and increased production costs [56,58–60] were observed in organic viticulture. Grape composition, wine quality and wine sensory characteristics are less influenced by the management regime [52,54,56,58,61–64]. Biodynamic viticulture showed reduced yields [56], a reduced ratio of yield:pruning weight [10] and reduced disease frequency of Botrytis [56] compared to organic viticulture. In a recent study, red wines from biodynamic production showed decreased alcohol content, decreased phenolic compounds, decreased wine color, decreased total polymeric pigments and decreased tannin concentration [65]. Soil quality [10], macronutrient supply in leaves [55,58], grape composition [10,56,63,64] and wine sensory characteristics [56,65] do not seem to be affected by biodynamic practices in comparison to organic viticulture.

However, there is a lack of research on the underlying mechanisms that induce changes in organically grown perennial crops [66]. Effects of consecutive years may overlap as[55] a consequence of the perennial growth habit of perennial crops. This makes the cause-effect relationship more complex. It might also explain the scarcity of studies dealing with the key factors responsible for the changes observed under the different management practices. The characterization of physiological processes of plants under different management systems can be helpful to understand the mechanisms that cause the changes. This is necessary to improve agricultural practices and to determine effective farming systems. Moreover, the effect of organic agriculture on food quality is still controversial and it is still unconfirmed whether organic agriculture has any beneficial effects on the product quality [3].

The aim of this study was to compare different management systems for vineyards including integrated, organic and biodynamic production according to the latest standards of the respective production systems in viticulture. Growth, yield and winegrape quality were determined for the different vineyard management systems over three consecutive seasons from 2010–2012. Beyond that, general principles responsible for the various effects of the different management systems were investigated. This included the detection of nutrient status, physiological performance and disease incidence. The study can potentially contribute to a better understanding of long-term effects of organic farming on growth, yield and fruit quality of grapevines. This knowledge is crucial to improve the respective management systems and to further develop sustainable cropping systems.

## Materials and Methods

### Experimental site

The field experiment was conducted in Geisenheim (49° 59′; 7° 56′). The experimental site was 0.8 hectare in size and planted in 1991 (*Vitis vinifera* L. cv. Riesling, clone Gm 198–30, grafted on *Vitis berlandieri* Planch. x *Vitis riparia* Michx. cv. SO4 and *Vitis riparia* Michx. x *Vitis cinerea* Engelm. cv. Börner rootstock, respectively). The experimental site is owned by Hochschule Geisenheim University.

The vines were planted at a spacing of 1.2 m within rows and 2 m between rows using a vertical shoot positioning system (VSP). Until the end of 2005 the vineyard was managed according to the code of *good practice* [67]. Conversion to organic and biodynamic viticulture started in 2006.

The experiment was set up as a complete block design, where the three factor levels of the main effect management system were replicated in four blocks. Each main plot for the factor management system was subdivided into two subplots, which were used for the two levels of the main effect rootstock. Each plot consisted of four rows with 32 vines each. Only the inner two rows of each plot were used for data collection. The outer rows were considered as buffer rows.

The plots were checked for uniformity prior to data collection using a balanced fixed factorial analysis of variance (with factors treatment, block) with respect to particle size distribution, soil moisture, pH, humus content, C/N ratio, and phosphor, magnesium and potassium content. Treatments did not differ significantly in any of these parameters (S1 Table).

Grape clusters of the respective treatments were analyzed for residues of systemic plant protection agents in 2009 to determine the impact of close neighborhood of integrated and organic plots on residue levels [68]. Active agents were investigated on clusters by GS-MS in Landesbetrieb Hessisches Landeslabor using an official protocol for residue detection [69]. No residues of systemic plant protection agents used in the integrated pest management could be found in the organic plots adjacent to the integrated plots (S2 Table). Therefore the plot size was considered suitable for detecting effects of the respective management system. The level of active agents found on clusters from integrated plots were below the maximum residue level (S2 Table) [70].

A weather station located approximately 500 m from the trial site was used for climate data collection. Data of weather conditions during the three seasons 2010 to 2012 are provided in S1 Fig. Long term annual rainfall for the site is 540 mm [71]. Total rainfall in the three seasons 2010–2012 was 659 mm, 469 mm and 531 mm, respectively. Growing season rainfall was 426 mm, 306 mm and 330 mm for the seasons 2010–2012, respectively.

### Management

The integrated treatment was managed according to the *code of good practice* [67]. Organic and biodynamic plots were managed according to Regulation (EC) No 834/2007 [72] and Regulation (EC) No 889/2008 [73] and according to ECOVIN- and Demeter-Standards, respectively.

All three treatments received compost during the period of conversion. After analysis of the composts the same amount of nitrogen equivalents were applied to every treatment. Green waste compost was used for the integrated plots and farmyard manure for the organic and biodynamic plots. In addition, biodynamic compost preparations 502–507 were applied to the compost for the biodynamic plots.

Both, organic and biodynamic treatments received identical soil and vine management practices except that biodynamic preparations were only applied to the biodynamic plots. The Wolff-Mixture® was used as cover crop (S3 Table) in both, the organic and biodynamic plots. Nitrogen supply of the organic and the biodynamic treatment was ensured by breaking up and tilling under the cover crop mixture (rich in legumes) of every second row shortly before full-bloom. In the integrated plots a grass mixture was established as cover-crop in between the rows. Every second row was ploughed shortly before bloom together with the cover crop of the organic and the biodynamic treatments. The integrated plots are amended with mineral fertilizers exclusively (50 kg N*ha^-1^*a^-1^ on 06/26/10, one day after full-bloom and 25 kg N*ha^-1^*a^-1^ on 07/05/12, six days after full-bloom) to compensate for the nitrogen introduction in the organic and the biodynamic treatment that occurred due to the ploughing of the cover crop rich in legumes.

In the organic and the biodynamic treatments mechanical under-vine management was implemented. In the integrated plots weeds in between the vines were controlled by herbicides.

*Erysiphe necator* and *Plasmopara viticola* (powdery and downy mildew) were controlled by applying systemic fungicides in integrated viticulture. Bitter salts MgSO_4_ were applied in the integrated treatment on 08//13/10, 07/11/11 and 07/26/11 and magnesium nitrate fertilizer was applied on 08/02/12 and 08/14/12. Botryticides were applied twice a year (S4 Table). For disease control in the organic and the biodynamic treatments copper, sulfur, and plant strengtheners (Mycosin VIN®, sodium bicarbonate, sodium silicate) were used to control powdery and downy mildew (S5 Table). In all treatments RAK® 1+2 M (500 dispensers*ha^-1^; 178 mg of (E,Z)-7,9-Dodecadienylacetate per dispenser and 205 mg of (Z)-9-Dodecenylacetate per dispenser) was applied against the vine moth and the European grapevine moth (*Eupoecilia ambiguella* and *Lobesia botrana*) following the mating disruption method.

The biodynamic field spray preparations horn manure and horn silica were each applied three times a year. Horn manure was applied once after harvest and twice in spring and horn silica was applied at grapevine phenological stages shortly before full-bloom, at veraison and shortly before harvest. In case no compost was applied to the biodynamic plots, the cow pat pit preparation was applied once a year in the growing season in parallel with tillage.

An overview of the management of the different treatments is given in Table 1.

**Table 1:** Overview of the management of the different management systems in this study.

Management practice	biodynamic	organic	integrated
cover crop	Wolff-mixture		grass mixture
under-vine-management	mechanically		herbicides
fertilization	ploughing up cover crop + compost with biodynamic preparations	ploughing up cover crop + compost	mineral fertilizers + compost
plant protection	copper + sulfur + plant strengtheners		systemic fungicides
biodynamic preparations	horn manure, horn silica, compost preparations	-	-

### Growth

Phenological stages were determined according to Coombe [74]. For this purpose 15 organs, i.e. buds, shoots or bunches per row were taken into account. Lateral leaf area was measured non-destructively at veraison. In 2010 the model of Lopes and Pinto [75] was applied, in 2011 and in 2012 the model of Mabrouk and Carbonneau [76] was applied. Both models have been shown to be applicable for estimating lateral leaf area of *Vitis vinifera* cv. Riesling under different management systems [77]. The calibration equations adapted to *Vitis vinifera* cv. Riesling were used for the respective models. Lateral leaf area of 6 shoots (2010) and 9 shoots (2011 and 2012) per row was determined on 2 and 3 vines, respectively, measuring lateral leaf area of one primary shoot at the beginning of the cane, one in the middle and one at the end of the cane. Whole-plant lateral leaf area was obtained by multiplying the secondary leaf area per shoot with the average number of shoots per vine. Leaf area index (LAI) was estimated in 2012 using the Plant Canopy Analyzer (PCA, LAI-2200, LI-COR, Lincoln, NE, USA) as described by Döring et al. [78]. Four measurements per treatment were carried out on 09/05/12, one in each block comprising eight vines on each side of two adjacent rows. Pruning weight of every vine of the central rows was determined gravimetrically in all three growing seasons. Relative levels of total chlorophyll in leaves were estimated at full-bloom, veraison and before harvest in the three growing seasons 2010 to 2012 using a portable chlorophyll meter (SPAD-502, KONICA MINOLTA INC., Tokyo, Japan). SPAD values are highly correlated to Chlorophyll content [79,80]. Nine mature, non-senescent leaves per row with comparable plastochron indices [81] were measured and three measurements per leaf were done (base, middle, leaf tip).

### Nutrient Status

Mineralized soil nitrogen content (Nmin) was measured at the phenological stages of full-bloom, pea-sized berries and shortly before harvest. Four samples per row in two depths (0–30 cm and 30–60 cm, respectively) were taken with a soil core sampler. Two rows per management system in each plot were sampled and analyzed separately. Samples were homogenized with a soil homogenizer (Schäfer, Euskirchen, Germany). Samples were analyzed according to Schaller [82] by flow injection analysis at 540 nm using a FOSS Tecator FIAstar Analyzer (FOSS, Hillerød, Denmark).

Nitrogen, phosphorus, magnesium and potassium content in grapevine tissue were measured at full-bloom and veraison during the three seasons, as recommended by Robinson [Robinson 2006]. For this purpose ten healthy leaves per row opposite to the first inflorescence or the first cluster of a shoot were picked. The leaf blade was washed with distilled water, dried at 60°C, ground to a fine powder by Foss Cyclotec™ 1093 (FOSS, Hillerød, Denmark). 0.25 g of the ground leaf tissue was used for the wet decomposition procedure. The samples were digested for 1 ½ hours at 100°C with 10 mL of a mixture of 420 mL H_2_SO_4_ conc., 330 mL 30% H_2_O_2_, 0.48 g selenium and 14 g Li_2_SO_4_ according to Schaller [82]. Samples were analyzed by inductively coupled plasma with optical emission spectroscopy (ICP-OES, Spectro Arcos, Spectro Analytical Instruments GmbH, Kleve, Germany). Standard curves were obtained using a multi-element standard solution Multielement-Standardlösung “Stammlösung Blatt” 8 Elemente in Salpetersäure 1 mol*L^-1^ (Bernd Kraft GmbH, Duisburg, Germany). Individual readings are the average of two measurements and varied by less than 5%. Nitrogen in the leaf tissue was analyzed by flow injection analysis using a FOSS Tecator FIAstar 5000 Analyzer (FOSS, Hillerød, Denmark).

### Physiological Performance

Leaf gas exchange measurements [net assimilation (*A*) and transpiration rate (*E*)] and stomatal conductance measurements [*g*_*s*_] were carried out on mature, non-senescent leaves with comparable leaf plastochron indices [81] on sunny days between 9 to 12 am. The leaves selected were well-exposed to direct sunlight (PAR >1000 μmol m^−2^s^-1^). Gas exchange was measured using an open gas exchange system (GFS 3000, Walz, Effeltrich, Germany). Pre-dawn water potential [Ψ_pd_] was determined in 2011 and 2012 on mature, undamaged and non-senescent leaves using a pressure chamber [83] (Soilmoisture Corp., Santa Barbara, CA, USA) according to Turner [84]. Prior to the measurements leaves were wrapped in polyethylene bags and detached from the shoot with a single cut.

### Yield

Crop yield was determined gravimetrically at harvest on 10/13/10, 09/20/11 and 10/10/12, respectively, on all vines in the plot except the buffer rows. Leaf area to fruit weight ratio [85] was determined in 2012 using LAI-measurements for leaf area estimation of the whole canopy and crop yield, as described above. Cluster weight [g], cluster length [cm] and cluster compactness index [g cm^-2^] were determined before veraison in 2012. Three healthy clusters per row (first clusters) were randomly selected and analyzed for cluster weight and cluster length. Cluster compactness was calculated as the ratio of cluster weight [g] to cluster length squared [cm^2^] according to Tello and Ibáñez [86].

The percentage of yield difference in the organic and the biodynamic treatments compared to the integrated management was calculated. The influence of berry weight, cluster weight and disease incidence and severity of downy mildew on yield reduction was estimated. Data of average single berry weight shortly before harvest, disease frequency of downy mildew before veraison and cluster weight at veraison in 2012 were used to estimate the influence of these parameters on yield reduction in the organic and the biodynamic treatments.

### Disease Incidence and Severity

Since the infestation with downy mildew potentially decreases grapevine yield, disease incidence and severity on clusters was monitored on 07/15/10, 07/01/11 and 07/13/12, respectively, according to organization Eampp guidelines [87]. For this purpose 100 clusters per row were used for estimation of disease severity, 50 on each side of the canopy. Disease incidence and severity were rated on a scale of 1 to 7, where 1 corresponds to no disease and 7 corresponds to 75–100% disease.

Infestation with Botrytis on clusters was determined shortly before harvest on 10/08/10, 09/19/11 and 10/09/12, respectively, following the Eampp guidelines [87] mentioned above. For this purpose 100 clusters per row were used for estimation of disease severity, 50 on each side of the canopy. In parallel with the determination of infestation with Botrytis disease frequency of sour rot on clusters was detected shortly before harvest.

### Winegrape Quality

Representative maturity samples (100 berries per row on each date) were collected to determine fruit quality parameters. Mean single berry weight of the samples was determined gravimetrically. The juice of the samples was obtained by pressing the berries with a sampling press at 1 bar (Longarone 85, QS System GmbH, Norderstedt, Germany) for two minutes the day after sampling. Maturity sampling took place every two weeks after veraison in 2010 and 2011 and every week after veraison in 2012. The concentration of α-amino-acids (N-OPA) in the juice was determined according to Dukes and Butzke [88]. α-amino acid groups were derivated with o-phthaldialdehyde/N- acetyl-L-cysteine (OPA/NAC) reagent. Absorbance at 335 nm was measured with a UV/VIS spectrometer (SPECORD 500, Analytik Jena AG, Jena, Germany) against a juice blank. Results were calculated as mg isoleucine equivalent from a standard curve. The must was analysed for soluble solids (°Brix) by refractometry and for total acidity and pH by Fourier-transform infrared spectroscopy (FTIR) (FT2 Winescan, FOSS, Hillerød, Denmark).

### Statistical Analysis

A balanced fixed factorial analysis of variance was carried out using the model

$$y=mu+s_{i}+r_{j}+b_{k}+q_{l}+{\left(sr\right)}_{ij}+{\left(sq\right)}_{il}+e_{ijkl}$$ (1)

where

mu is the mean,

s_i_ (i = 1..3) are the effects of the management system, r_j_ (j = 1,2) are the effects of the rootstock, b_k_ (k = 1..4) are the block effects, q_l_ (l = 1..3) are the year effects, and e_ijkl_ is a random error term. The effects (sr)_ij_ and (sq)_il_ are interactions between the corresponding main effects.

If a main effect or an interaction was significant (p<0.05), a Tukey test was carried out to compare the factor levels. Calculations were carried out with the AOV and Tukey`s HSD commands of the statistical software R [89]. For all the parameters measured averages per combination of treatment:rootstock:block (n = 1) were calculated and used for statistical analyses. For certain parameters that vary over time such as mineralized nitrogen content Nmin in the soil, assimilation rate *A*, transpiration rate *E*, stomatal conductance *g*_*s*_, pre-dawn water potential Ψ_pd_ and berry quality parameters during ripening the date was also included as a fixed factor into the model. For the parameter mineralized nitrogen content in the soil the factors soil management (cover crop or cultivated soil) and sampling depth (0–30 cm and 30–60 cm) were included into the model as a fixed factor. For parameters measured in just one season such as leaf area index (LAI) and cluster compactness parameters, the factor year and the interactions with the factor year were removed from the model. In case of LAI, the rootstock was not taken into account because data collection equally included the rootstocks Boerner and SO4 by measuring within a transect of two adjacent rows.

## Results

### Growth

Lateral leaf area differed significantly among treatments (Table 2). The integrated treatment showed the highest lateral leaf area with 4.26 m^2^ per plant and differed significantly from the other two treatments. The organic and the biodynamic management systems showed an average lateral leaf area of 3.45 m^2^ and 2.95 m^2^ per vine, respectively, and did not differ significantly from each other. Lateral leaf area differed significantly among years. 2012 showed a significantly lower leaf area compared to 2010 and 2011.

**Table 2:** Results of the balanced fixed factorial analysis of variance (ANOVA) and results of the Tukey`s test for the fixed factor management system.

field of interest	parameter	treatment	int (means ± se)		org (means ± se)		biodyn(means ± se)		rootstock	block	year	date	interaction treatment:rootstock	interaction treatment:year	soil management	depth
growth	lateral leaf area [m^2^] per vine	***	4.26 ± 0.25	a	3.45 ± 0.29	b	2.95 ± 0.23	b	ns	ns	***	-	ns	ns		
	Leaf area index (LAI) in 2012	***	2.44 ± 0.11	a	1.64 ± 0.07	b	1.72 ± 0.07	b	-	ns	-	-	-	-		
	pruning weight [dt * ha^-1^]	***	44.9 ± 1.77	a	38.5 ± 0.97	b	37.4 ± 1.38	b	***	**	***	-	*	***		
	relative chlorophyll content full-bloom	Ns	38.9 ± 0.45	-	38.2 ± 0.37	-	38.2 ± 0.31	-	***	*	***	-	ns	**		
	relative chlorophyll content veraison	***	42.9 ± 0.43	a	41.5 ± 0.42	b	40.9 ± 0.47	b	**	**	***	-	ns	*		
	relative chlorophyll content harvest	**	41.6 ± 0.73	a	40 ± 0.68	b	40.4 ± 0.75	b	**	**	***	-	ns	ns		
nutrient status	Nmin [kg*ha^-1^] in soil	***	14.37 ± 1.67	b	21.44 ± 1.69	a	20.78 ± 1.54	a	-	ns	***	***	-	***	***	***
	nitrogen content in leaves [%] full-bloom	Ns	2.8 ± 0.03	-	2.77 ± 0.04	-	2.75 ± 0.04	-	**	ns	***	-	ns	*		
	nitrogen content in leaves [%] veraison	*	2.1 ± 0.04	b	2.16 ± 0.04	a	2.16 ± 0.04	a	*	***	***	-	ns	*		
	magnesium content in leaves [%] full-bloom	ns	0.22 ± 0.007	-	0.22 ± 0.004	-	0.21 ± 0.005	-	ns	***	***	-	ns	ns		
	magnesium content in leaves [%] veraison	*	0.23 ± 0.008	a	0.22 ± 0.005	ab	0.21 ± 0.007	b	**	***	ns	-	ns	ns		
physiologicalperformance	assimilation rate A [μmol CO_2_ m^-2^ s^-1^]	***	10.3 ± 0.35	a	8.5 ± 0.3	b	8.4 ± 0.32	b	ns	*	***	***	ns	*		
	transpiration rate E [mmol m^-2^ s^-1^]	***	2.42 ± 0.12	a	1.98 ± 0.1	b	1.98 ± 0.1	b	**	*	***	***	ns	ns		
	stomatal conductance g_s_ [mmol H_2_O m^-2^ s^-1^]	***	117.84 ± 4.77	a	92.72 ± 3.88	b	90.27 ± 3.99	b	ns	***	***	***	ns	ns		
	pre-dawn water potential Ψ_pd_ [MPa]	**	-0.2 ± 0.01	a	-0.21 ± 0.01	a	-0.23 ± 0.01	b	***	***	***	***	ns	*		
yield	yield [kg * ha^-1^]	***	6984 ± 559	a	4276 ± 302	b	4347 ± 287	b	ns	ns	***	-	ns	***		
	leaf-area-to-fruit-weight-ratio [cm^2^ * g^-1^] in 2012	Ns	25.11 ± 0.85	-	32.41 ± 3.2	-	32.94 ± 4.08	-	-	ns	-	-	-	-		
	average single berry weight [g]	***	see Fig 4	a	see Fig 4	b	see Fig 4	b	***	ns	ns	***	ns	ns		
	cluster weight [g] at veraison in 2012	**	122.29 ± 7.46	a	101.94 ± 9.37	b	91.92 ± 3.37	b	ns	*	-	-	*	-		
	cluster length [cm] at veraison in 2012	ns	10.2 ± 0.34	-	10.02 ± 0.47	-	9.68 ± 0.28	-	ns	*	-	-	ns	-		
	cluster compactness index [g*cm^-2^] at veraison in 2012	*	1.17 ± 0.04	a	1 ± 0.03	b	0.99 ± 0.05	b	ns	ns	-	-	ns	-		
disease incidenceand severity	disease incidence/severity *Plasmopara viticola*	***	1.02 ± 0.01	b	2.02 ± 0.17	a	1.89 ± 0.15	a	ns	ns	***	-	ns	***		
	disease incidence/severity *Botrytis cinerea*	**	4.49 ± 0.39	b	4.64 ± 0.41	ab	4.82 ± 0.42	a	ns	***	***	-	ns	*		
	disease incidence sour rot [% infested clusters]	***	6.17 ± 1.42	a	0.83 ± 0.29	b	0.62 ± 0.29	b	ns	ns	***	-	ns	***		
winegrape quality	total soluble solids [°Brix]	ns	see S2 Fig	-	see S2 Fig	-	see S2 Fig	-	ns	ns	ns	***	ns	ns		
	total acidity [g*L^-1^]	ns		-		-		-	ns	ns	***	***	ns	ns		
	pH	ns		-		-		-	ns	ns	***	***	ns	ns		
	N-OPA	**	see Fig 4	b	see Fig 4	ab	see Fig 4	a	*	**	***	***	ns	***		

*, ** and *** indicate statistical significance (p<0.05; p<0.01 and p<0.001) of the main effects determined by ANOVA (ns = not significant). Different letters indicate statistically significant differences (p<0.05) for the fixed factor management system determined by the Tukey`s test (int = integrated treatment, org = organic treatment, biodyn = biodynamic treatment).

LAI assesses whole plant leaf area which is influenced by both main shoot and lateral leaf area. It differed significantly among treatments on 09/05/12 shortly before harvest. The integrated treatment again showed the highest LAI value of 2.44 and differed significantly from the other two treatments.

**Fig 1:**
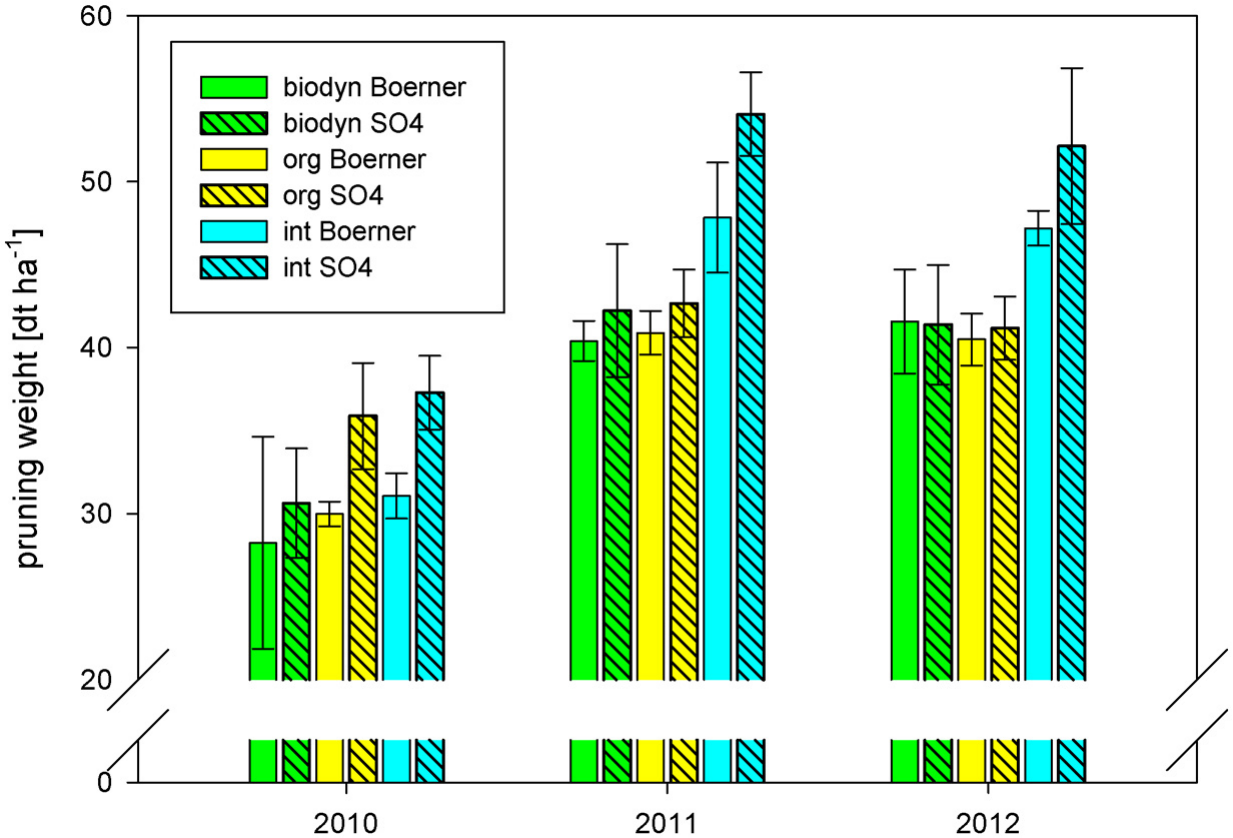
Pruning weight [dt ha^-1^] from 2010–2012. Means ± sd per management system, year and rootstock (int = integrated treatment, org = organic treatment, biodyn = biodynamic treatment).

Another important parameter for vigor is the pruning weight. The integrated treatment showed a significantly higher pruning weight compared to the organic and the biodynamic treatments (Fig 1). Average pruning weight for the integrated management system was 44.9 dt ha^-1^, while the organic and the biodynamic treatments showed 38.5 dt ha^-1^ and 37.4 dt ha^-1^, respectively. The rootstock, block, and year had a significant effect on pruning weight (Table 2). Interactions between treatment and rootstock occurred. Boerner showed lower pruning weight compared to SO4 except for the biodynamic treatment in 2012. Interactions between treatment and year occurred, because the biodynamic treatment showed the lowest pruning weight except for 2012 where the organic management system showed the lowest value (Fig 1).

Relative levels of total chlorophyll index did not differ significantly among treatments at full-bloom, but later in the season it differed significantly among treatments. The two biological systems showed significantly lower chlorophyll index compared to the integrated treatment at veraison and harvest, respectively (Table 2). Interactions between treatment and year occurred concerning relative levels of total chlorophyll index at veraison. The integrated treatment showed the highest values except for 2012 where the organic plots showed the highest relative levels of total chlorophyll. The biodynamic plots showed the lowest relative levels of total chlorophyll except for 2010 where the organic treatments showed lower levels.

### Nutrient Status

The organic and the biodynamic treatments showed a significantly higher mineralized nitrogen content in the soil compared to the integrated management system. The integrated treatment was fertilized with mineral fertilizers exclusively from 2010 to 2012 to compensate for the nitrate introduction by the cover crop used in the organic and the biodynamic plots. The organic and the biodynamic treatments both showed average nitrogen levels of 20 kg ha^-1^, whereas the integrated treatment showed just an average nitrogen level in the soil of 14 kg ha^-1^. The organic and biodynamic treatments did not differ significantly in the content of mineralized nitrogen during the growing seasons 2010 to 2012 (Table 2). The year, the date, the sampling depth and the tillage system significantly influenced the nitrate content in the soil. 2012 showed a significantly higher content of mineralized nitrogen in the soil compared to 2010 and 2011, respectively. The tilled rows showed higher nitrogen content compared to the rows where cover crop was established during the growing season. In the upper soil layer (0–30 cm) there was significantly more mineralized nitrogen present than in the lower layer (30–60 cm). Interactions between treatment and year were observed. The organic treatment showed the highest levels of mineralized nitrogen in the soil except for the season 2012 where the biodynamic management system showed the highest levels.

Nitrogen and magnesium content in leaves did not differ significantly among treatments at full-bloom.

In contrast, nitrogen and magnesium content differed significantly among treatments at veraison in all three growing seasons (Table 2). The integrated treatment showed significantly lower nitrogen content in leaves at veraison, but an interaction between treatment and growing season was observed. The organic treatment showed the highest nitrogen content in the leaf tissue in 2010, whereas the biodynamic treatment showed the highest values of nitrogen in the leaf tissue in 2012. In the dry season 2011 nitrogen contents in the leaf tissue of all treatments were similar. When compared to the biodynamic system, the integrated treatment showed significantly higher magnesium content in leaves at veraison.

### Physiological Performance

**Fig 2:**
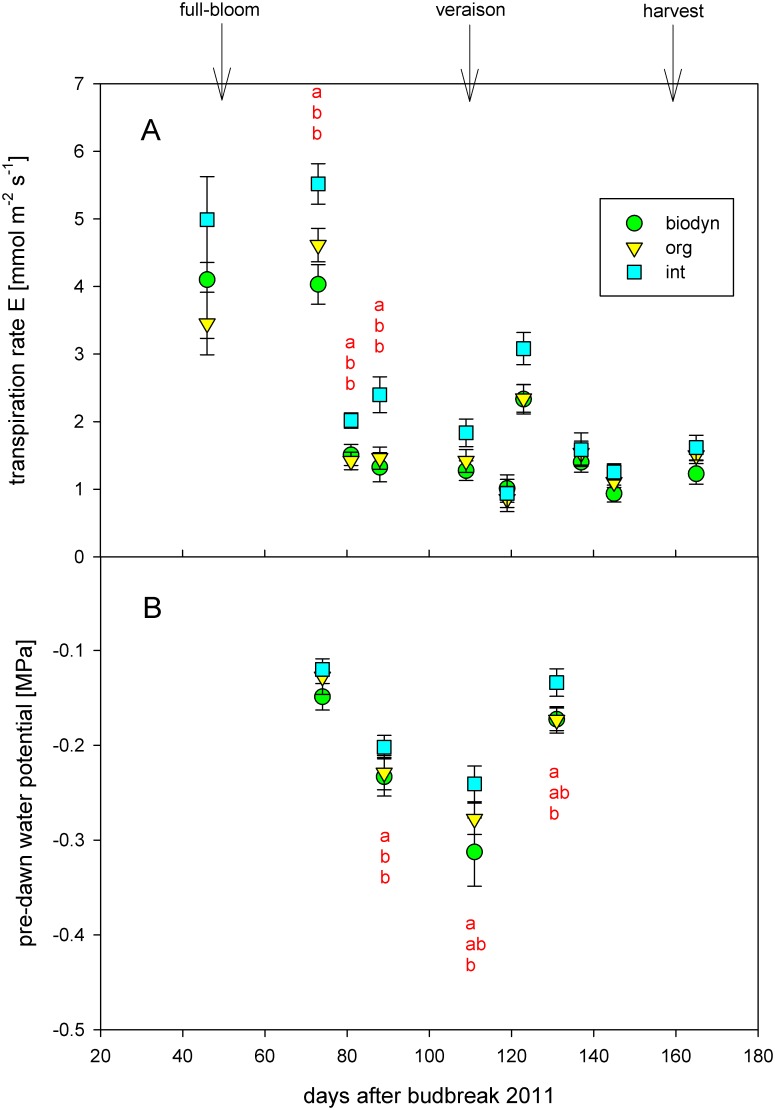
(A) Transpiration rate E [mmol m^-1^ s^-1^] and (B) pre-dawn water potential Ψ_pd_ [MPa] in 2011. Means ± se per management system. Different letters indicate statistically significant differences (ANOVA and Tukey`s test, p<0.05) for the specific date. Arrows indicate full-bloom, veraison and harvest, respectively (int = integrated treatment, org = organic treatment, biodyn = biodynamic treatment).

Assimilation rate *A*, transpiration rate *E* and stomatal conductance *g*_*s*_ differed significantly among treatments in the three growing seasons 2010 to 2012. Organic and biodynamic treatments showed significantly lower assimilation rate, transpiration rate and stomatal conductance compared to the integrated treatment. The mentioned parameters also differed significantly among years, blocks, and dates. The transpiration rate differed significantly between rootstocks. Boerner showed a significantly higher transpiration rate in comparison to SO4 for all treatments. An interaction between treatment and year occurred in the case of assimilation rate. The biodynamic treatment showed higher assimilation rates than the organic treatment in 2011, but showed lower assimilation rate A than the organic treatment in 2011 and in 2012 (Table 2). The development of the transpiration rate E during the growing season 2011 is shown in Fig 2. The differences in transpiration rate E among the treatments were the highest after full-bloom.

Pre-dawn water potential (Ψ_pd_) was measured in 2011 and 2012. It significantly differed among treatments (Table 2). The biodynamic treatment showed a significantly higher level of water stress (lower pre-dawn water potential) compared to the integrated and the organic treatments. The rootstock, the season, the date, and the block also had a significant influence on the pre-dawn water potential. Boerner showed a significantly higher level of water stress compared to SO4. When individual seasons were compared, 2012 showed a significantly higher level of water stress compared to 2011. An interaction between treatment and year was detected for the pre-dawn water potential. The integrated treatment showed the lowest level of water stress in 2011 and the organic treatment showed the lowest level of water stress in 2012.

### Yield

**Fig 3:**
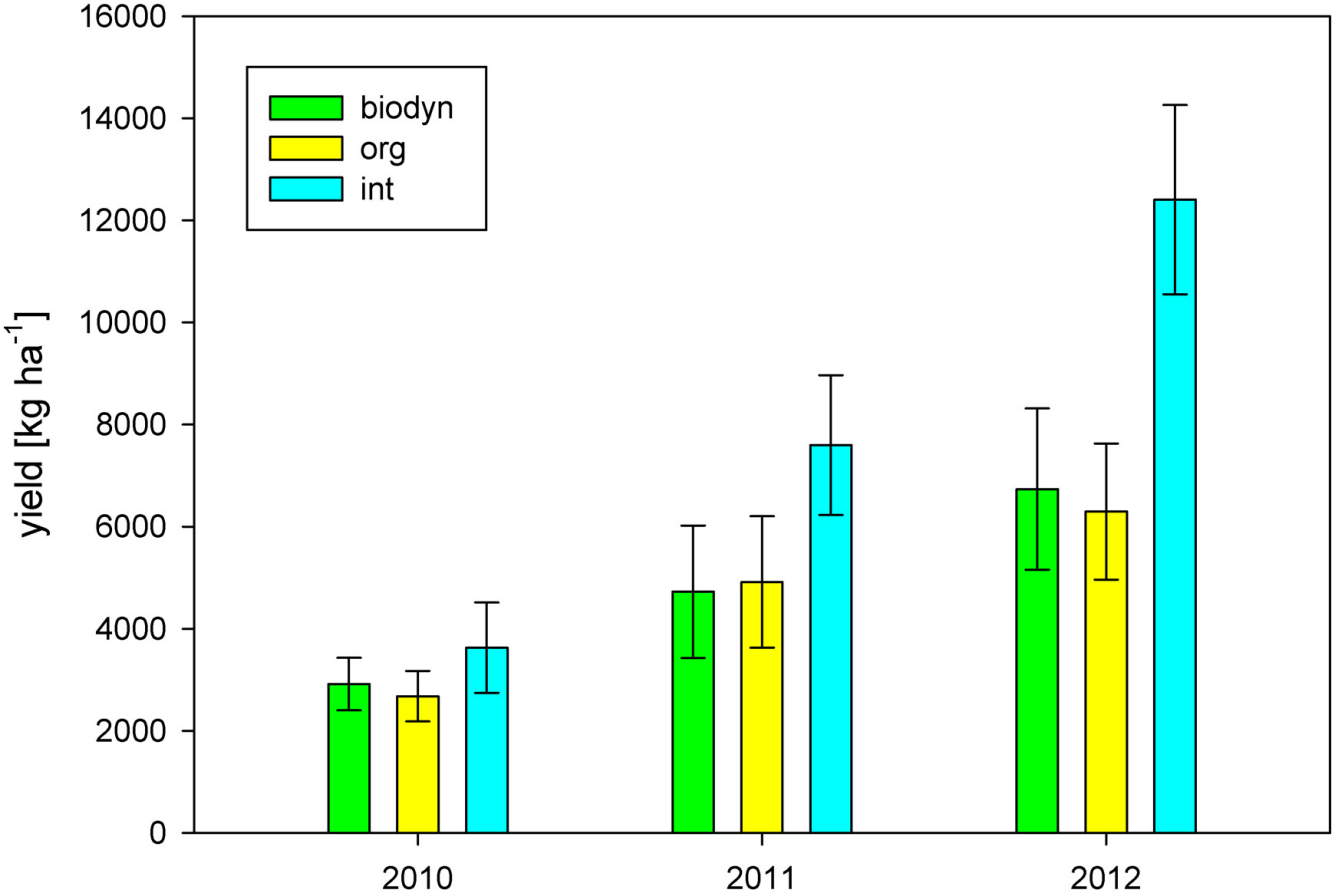
Yield [kg ha^-1^] from 2010–2012. Means ± sd per management system and year (int = integrated treatment, org = organic treatment, biodyn = biodynamic treatment).

Yield differed significantly among treatments and among years. The integrated treatment showed a significantly higher yield compared to the organic and the biodynamic treatments across the three growing seasons 2010 to 2012 (Table 2). Average yield of the integrated management system was 6984 kg ha^-1^, whereas yields of the organic and the biodynamic management systems were 4276 kg ha^-1^ and 4347 kg ha^-1^, respectively. 2010 showed the lowest average yield. Interactions between the factors treatment and year were recorded. The organic treatment showed the lowest yield in 2010 and 2012 and the biodynamic treatment showed the lowest yield in 2011 (Fig 3).

Leaf area to fruit weight ratio is a major indicator for vine balance of vegetative and reproductive performance. In 2012, it did not differ significantly among treatments (Table 3). The integrated treatment showed a leaf area to fruit weight ratio of 25.11 cm^2^ g^-1^ on average. Both, the organic and the biodynamic treatment showed a slightly increased average leaf area to fruit weight ratio of 32.41 cm^2^ g^-1^ and 32.94 cm^2^ g^-1^, respectively.

**Table 3:** Average values of estimated yield reduction of the organic and the biodynamic treatment compared to the integrated treatment.

		2010	2011	2012
org	gravimetrically measured yield reduction [%] at harvest	26.2	35.3	46.2
	estimated yield reduction caused by downy mildew [%]	11.2	0	6
	estimated yield reduction caused by berry weight [%]	5.9	8.5	1.1
	estimated yield reduction caused by bunch weight [%] at veraison	-	-	16.6
biodyn	gravimetrically measured yield reduction [%] at harvest	19.6	37.8	44.5
	estimated yield reduction caused by downy mildew [%]	10.3	0	3.2
	estimated yield reduction caused by berry weight [%]	2.7	8.5	1
	estimated yield reduction caused by bunch weight [%] at veraison	-	-	24.8

Yield reduction [%] is calculated from gravimetrically measured yield at harvest, yield reduction by downy mildew is estimated according to EPPO-guideline, yield reduction by berry weight is estimated taking into account average berry weight before harvest and yield reduction by cluster weight is estimated according to differences in cluster weight at veraison in 2012 (org = organic treatment, biodyn = biodynamic treatment).

**Fig 4:**
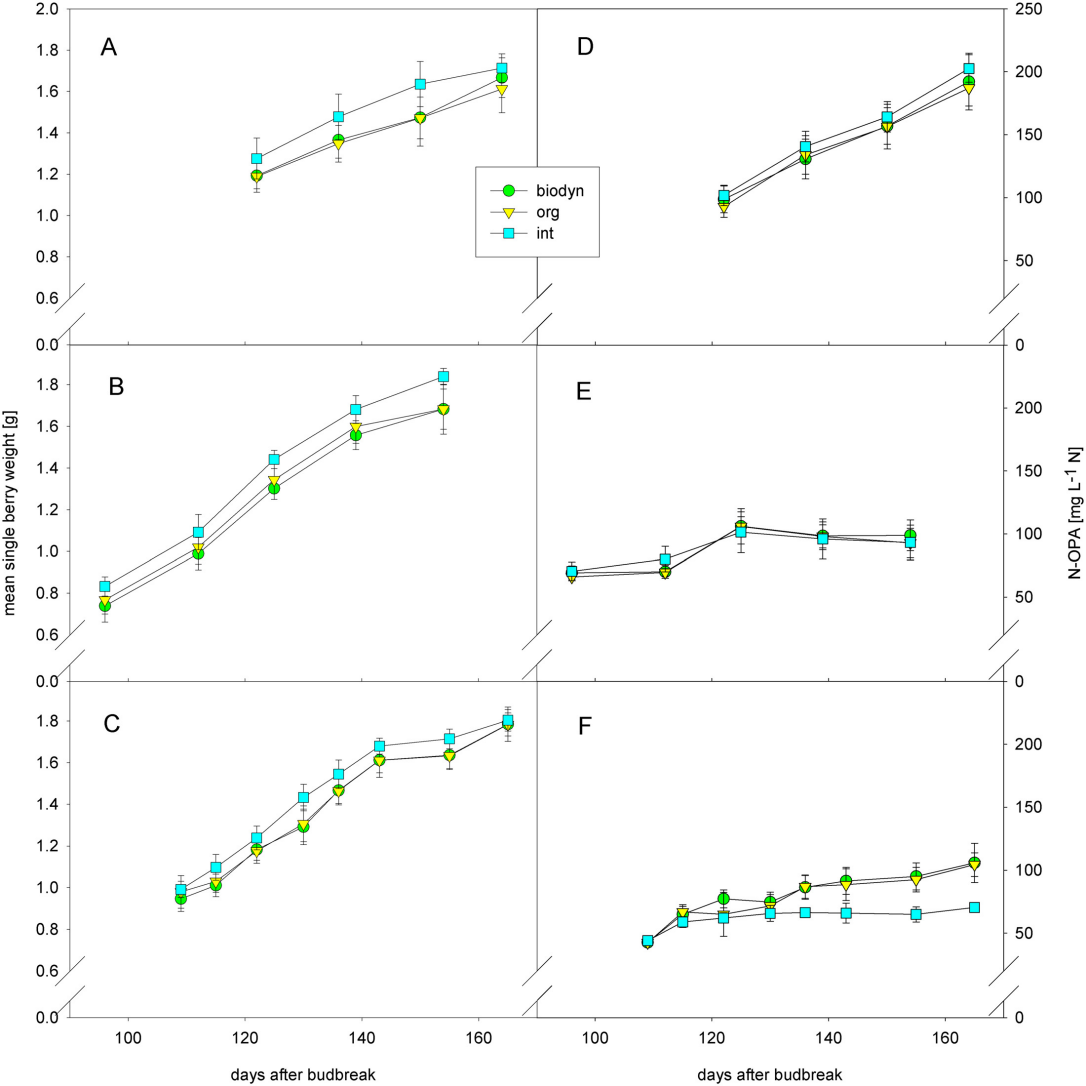
Mean single berry weight [g] in (A) 2010, (B) 2011, (C) 2012 and α-amino acid content (N-OPA) [mg L^-1^ N] in (D) 2010, (E) 2011 and (F) 2012. Means ± sd per management system and year (int = integrated treatment, org = organic treatment, biodyn = biodynamic treatment).

Average single berry weight during ripening differed significantly among treatments. The integrated treatment showed a significantly higher berry weight. Average single berry weight was also influenced by the sampling date during ripening and the rootstock. Boerner showed a significantly lower berry weight compared to SO4 (Fig 4A–4C).

Average cluster weight at veraison differed significantly among treatments in 2012. The integrated treatment showed significantly higher cluster weight (122.29 g) compared to the organic and the biodynamic management. Under organic and biodynamic management, average cluster weights were 101.94 g and 91.92 g, respectively. Interactions between treatment and rootstock occurred. The integrated treatment showed the highest average cluster weight for Boerner and the organic treatment showed the highest average cluster weight for SO4. Cluster length of representative clusters did not differ significantly among treatments in 2012. Cluster compactness was assessed as the quotient of cluster weight and cluster length. The integrated treatment showed significantly higher cluster compactness compared to organic and biodynamic management (Table 2).

### Disease Incidence and Severity

The monitoring results for the infestations of downy mildew, a heterothallic oomycete, on grapes after flowering in 2010 and 2012 showed a significantly higher rate of infection in the organic and the biodynamic treatments, whereas hardly any infection of downy mildew on grapes of the integrated treatment was recorded (Table 2). The organic and the biodynamic treatments showed an average rate of infection of 2.02 and 1.89, respectively. In the integrated treatment the average rate of infection observed was of 1.02. The year had a significant influence on infection of downy mildew. In the dry season of 2011, no symptoms in any of the treatments were observed. Therefore, an interaction between treatment and year occurred. The organic treatment had the highest disease incidence of downy mildew in 2010 and 2012, respectively.

Disease incidence of Botrytis in this study differed significantly between the integrated and the biodynamic treatments (Table 2). The biodynamic treatment showed a significantly higher infection rate with an average value of 4.82, whereas the integrated treatment showed an average infection rate of 4.49. The block and the year had a significant effect on the infestation with Botrytis. In 2010 and 2011 disease frequency of Botrytis was high compared to 2012. Interactions between treatment and year were observed. The integrated treatment showed the lowest infection rate in 2010 and 2011 and the organic treatment showed the lowest infection rate in 2012.

The integrated treatment showed a significantly higher disease frequency of sour rot compared to the other treatments. The year had a significant influence on the infection of sour rot. There was an interaction between treatment and year, as no sour rot was detected in any treatment in 2012 (Table 2).

### Winegrape Quality

α-amino-acid content (N-OPA) differed significantly among treatments during ripening. The biodynamic treatment showed significantly higher values compared to the integrated treatment. N-OPA also differed significantly among years, blocks, rootstocks and dates of the maturity sampling during ripening. There was a clear interaction between treatment and year (Fig 4D–4F). In 2011 and 2012 the biodynamic treatment showed the highest amount of α-amino-acids in berries during ripening and at harvest, whereas the integrated treatment showed the highest α-amino-acid content in 2010 where values were generally higher.

pH, total acidity [g L^-1^] and total soluble solids [°Brix] did not differ significantly among treatments (Table 2). All three parameters differed among dates during ripening and pH and total acidity differed among years (S2 Fig).

## Discussion

### Growth

Growth and vigor expressed as lateral leaf area, LAI during ripening, pruning weight, and relative chlorophyll content in leaves was evidently reduced under organic and biodynamic management. Leaf area of the organic and the biodynamic treatments was sufficient to provide an adequate assimilation surface, because for a vertical shoot positioning system as it was applied here LAI values of 1.5 up to 3 are within the desired range [90]. Pruning weight of all treatments ensured a sufficient growth [91]. Hofmann, Corvers, Kauer and Meißner [52–55] report a reduction in pruning weight of the organic plots in different trials comparing conventional and organic viticulture under the same climatic conditions. Granstedt and Kjellenberg [32] observe a reduced number of side stems of potato plants applying biodynamic compared to conventional agricultural practices. This is in accordance with the reduced lateral leaf area of the organic and biodynamic treatments in this study.

Nitrogen levels of all treatments at full-bloom and veraison were within the desired range [92,93]. The organic and the biodynamic treatments showed both higher nitrogen content in the soil [Nmin] and higher nitrogen content in the leaf tissue [%] at veraison. Therefore nitrogen content in the soil and in the leaf tissue cannot account for the reduction in growth and vigor of the organic and the biodynamic treatments. This is unexpected and might be due to the effect of legumes in the cover crop (Wolff-mixture) of the organic and the biodynamic treatments. Because the soil was generally tilled shortly before flowering, it might also explain why no differences in nitrogen content at full-bloom were observed among treatments. Even though the integrated treatment received the addition of mineral fertilizer, it showed significantly lower nitrogen content in the soil and lower nitrogen content in the leaf tissue at veraison in comparison to the other two treatments. Interactions between treatment and year reveal that the organic treatment showed higher nitrogen content in soil and leaf tissue at veraison in 2010 and 2011, where it also showed a higher pruning weight in comparison to the biodynamic treatment, whereas in 2012 the biodynamic plots showed higher nitrogen content in soil and leaf tissue at veraison as well as higher pruning weight in comparison to the organic treatment. In the case of the integrated treatment the nitrogen content in the soil and in the leaf tissue seems to be encoupled from vigor and pruning weight.

Observed magnesium levels are in the required range of 0.21 to 0.34% in the leaf tissue during the growing season [92]. Magnesium content in the integrated treatment is slightly higher compared to the organic and biodynamic systems at veraison. Bitter salts were applied in the integrated treatment at 08/13/10, 07/11/11 and 07/26/11 and magnesium nitrate fertilizer (Magnisal™) was applied at 08/02/12 and 08/14/12 (S4 Table). This might be an important parameter since magnesium is needed for chlorophyll composition. Since addition of magnesium in the integrated treatment occurred around veraison, this might be one reason why chlorophyll content did not differ among treatments at full-bloom. The integrated treatment showed both significantly higher magnesium content at veraison and significantly higher chlorophyll content at veraison and harvest.

Phosphorous and potassium contents in grapevine leaves under different management systems did not show any relevant differences in this study (data not shown).

Assimilation rate, transpiration rate and stomatal conductance are significantly higher in the integrated treatment in the three growing seasons 2010–2012. The changes in physiological performance of the organic and biodynamic plots especially under dry conditions after full-bloom in 2010 and 2011 (Fig 2) might account for the observed changes in growth and vigor. It can be deduced that the integrated treatment had higher whole-plant assimilation and transpiration, because it showed higher lateral leaf area and higher LAI as well as higher assimilation rate, transpiration rate and stomatal conductance. Interactions between treatment and year for the assimilation rate are similar to the interactions that occurred for the indirect chlorophyll content at veraison. The organic plot showed the lowest assimilation rates and the lowest indirect chlorophyll content at veraison in 2010, whereas the biodynamic treatment showed the lowest assimilation rates and the lowest indirect chlorophyll content at veraison in 2011 and 2012, respectively. These two parameters seem to be clearly linked.

One hypothesis is that the different types of cover crops used in this study influence water availability in the soil and thus physiological performance, growth and vigor and cause interactions with the root systems of the vines. Pre-dawn water potential, a good indicator for water stress under humid climatic conditions [94], was lower under organic and biodynamic management, although just the biodynamic treatment differed significantly and interactions between treatment and year occurred. Monteiro and Lopes [95] report a decrease of pruning weight due to cover cropping in the third year of a trial comparing cultivated soil to the application of cover crops. Lopes et al. [96] discovered the transpiration rates per unit leaf area of some cover crop species to be about three times as high as those measured on grapevine leaves. The vigor and growth of the grapevines may not only be influenced by the water uptake of the Wolff-mixture in comparison to the grass mixture, but nutrient competition between cover crop and vines may also influence its chlorophyll content and growth. Due to the different cover crops there might also be a different distribution of soil moisture and therefore root development of the vines might be influenced [95,97]. Other interactions between plants of the cover crop and vines may be held responsible for changes in growth and physiological performance. Another important factor that might influence growth and vigor of the different treatments in this study are plant growth regulators such as gibberellic acid, cytokinin and especially auxin that is involved in the lateral inhibition process. Maybe differences in the root system or the water availability in the soil might account for different levels of these plant growth regulators in plant tissues under differing management systems. Investigation of available soil water in the different treatments on one hand and xylem sap flow on the other hand might provide a better insight of the relation between water potential and physiological performance of the treatments. Differences in xylem or leaf anatomy under the different treatments as a reaction to different water availability or different root distribution in the soil might as well account for differences in growth and physiological activity.

Furthermore, copper used as active ingredient in spraying agents against downy mildew might possibly influence physiological performance of organically and biodynamically grown vines and thus growth [98,99]. In this study excessive copper exposure in soils cannot account for the changes observed among the different management systems, since copper content in the soil did not differ significantly when determined in 2012 [data not shown]. Amounts were of 73.8 to 75.5 mg kg^-1^ of soil and thus well below the copper contents in soils considered harmful for grapevines. Some studies confirm metabolic and physiological changes of *Vitis vinifera* leaves exposed to Bordeaux mixture containing copper sulphate [100,101]. However, amounts of copper applied against downy mildew in this study were low (maximum 500 g per spraying event; S5 Table). The possible impact of spraying agents containing copper used in this study (Funguran, Funguran Progress and Cuprozin; S5 Table) on physiological performance of leaves should be further investigated to determine to which extend it impacts vine growth in comparison to systemic fungicides.

### Yield

Yield was significantly reduced under organic and biodynamic management in this trial. When yield was compared to the average yield of the growing area [102], the yield of the experimental trial followed similar patterns. According to previous studies yield under organic management seems to decrease except for legumes and perennial fruits such as apple, pear and peach. For most of the studies conducted in viticulture a yield decrease under organic management was observed [52–57]. The system in this study is also a legume-based system in which nitrogen supply of the organic and biodynamic treatments is ensured by a cover crop rich in legumes. The nitrogen supply of the vines cannot account for the observed yield differences because the biological systems showed higher nitrogen contents in the soil during the three growing seasons 2010–2012 and higher nitrogen content in leaves at veraison. No yield differences between the organic and the biodynamic system in our study were observed. Danner [56] reports a decrease of yield under biodynamic compared to organic management. This could not be confirmed here.

In 2012, cluster weight and cluster compactness were significantly reduced under organic and biodynamic management. Differences in nutrient supply, physiological performance, vigor as well as water availability may have caused these differences. Reproductive development of *Vitis vinifera* is highly sensitive to vine water status [103]. Water deficits early in the season were shown to result in decreases in yield and cluster weight. If early season water deficit occurred over two or more years, the number of grape clusters per vine and the cluster weight were reduced and both factors contributed to yield decreases [103]. In this study, two of the three consecutive seasons showed a decreased transpiration rate in the organic and the biodynamic treatments, especially between bloom and veraison (Fig 2). This decreased transpiration rate might have contributed to the reduction of cluster weights in the respective treatments. The period from initiation to maturation of the grape encompasses two growing seasons [104,105]. This is why water deficits may simultaneously affect more than one reproductive process and influence not only cluster weight, berry weight and yield of one year, but also primordia that highly determine yield of the subsequent growing season. It might be one reason for lower cluster weight and lower cluster compactness in the organic and the biodynamic treatments and might simultaneously have influenced yield of the respective subsequent year. Cluster number might be another very important parameter to better understand the reasons and mechanisms of the yield differences in the different management systems.

Berry weight differed significantly among treatments. The integrated treatment showed significantly higher average single berry weight during ripening and at harvest compared to the organic and the biodynamic treatments. This is in accordance with Linder [58] (*Vitis vinifera* L. cv. Chasselas), Pool and Robinson [57] (*Vitis labrusca* cv. Elvira), and Meißner [55] (*Vitis vinifera* L. cv. Riesling), who also detected a reduction in berry weight under organic viticulture. Concerning other crops, organically grown tomatoes also showed smaller mass [25]. Differences in berry weight among treatments were most evident in the dry year 2011. This might be due to lower leaf gas exchange of the organic and the biodynamic treatments after full-bloom [106]. Since there is evidence that water deficit during the period after flowering severely reduces berry weight in grapevines [107,108], it might account for the reduced berry weights observed in the organic and the biodynamic treatments, respectively.

In 2010 and 2012, it was observed that the plots under organic and biodynamic management displayed a higher disease incidence of downy mildew with an increased severity. This could primarily be due to the use of copper and plant strengtheners in the organic and the biodynamic plots (S5 Table) as opposed to the systemic fungicides that were applied in the integrated treatment (S4 Table). Danner [56] did not observe differences in disease frequency of downy mildew among integrated, organic and biodynamic viticulture in Austria (*Vitis vinifera* L. cv. Grüner Veltliner) from 1979–1983. In that study wettable sulfur, extracts of horsetail (*Equisetum arvense)*, valerian (*Valeriana officinalis*) and stinging nettle (*Urtica dioica*), alkali silicates (water glass) and calcium oxide (extracted from algae) were used as plant protection agents and plant strengtheners in the organic and the biodynamic treatments, whereas in this study wettable sulfur, water glass, Vitisan, copper and Mycosin VIN were applied (S5 Table).

Interactions between treatment and year for the parameters yield and disease incidence of downy mildew show similar patterns. In the two growing seasons 2010 and 2012, where downy mildew occurred, the organic management system showed higher disease incidence and lower yields than the biodynamic treatment. In 2011, where downy mildew was not detected in any of the systems, the biodynamic treatment showed the lowest yields.

Compared to the integrated treatment average yield reduction is 35.9% in the organic and 34% in the biodynamic treatment (Table 3). These yield reductions can be partially explained by the reduced cluster weight, the reduced berry weight and the increased disease frequency of downy mildew. Disease frequency of downy mildew and reduced cluster weight can account for 28% out of 44.5% of yield loss in the biodynamic treatment and can account for 22.6% out of 46.2% of the yield loss in the organic treatment in 2012.

Disease frequency of downy mildew, single berry weight and cluster weight cannot account for the entire yield reduction in the organic and the biodynamic treatments which occurred from 2010–2012 (Table 3). One weakness of this assessment may be that disease frequency of downy mildew was estimated at bunch closure and not shortly before harvest. The former assessment time was chosen because the shriveling of infested bunches make detection of the disease more difficult later in the season. In comparing the season 2010 to 2012, it was observed that in 2012 a higher yield loss in the biological systems occurred. In 2010 the infestation of downy mildew took place much earlier in the growing season. We can therefore deduce that more compensation occurred and that we detected a similar rate of infection as in 2012, but observed less yield reduction. The reduced cluster weight of the organic and the biodynamic treatments measured at veraison in 2012 can be partially held responsible for the yield reduction of the respective systems. We do not know if the number of bunches per shoot were similar among the management systems and we cannot quantify the yield loss due to Botrytis shortly before harvest. These two factors may highly determine yield of the management systems. Number of clusters per shoot as well as average cluster weight, average number of berries per cluster, average berry weight and average number of shoots per vine should be determined in the future to provide a more precise idea of the reproductive growth cycle in the different treatments.

### Winegrape Quality

Winegrape quality encompasses not only berry chemical traits, but also health status of the grapes and nutrient contents for ensuring successful yeast nutrition [109]. No differences in berry quality parameters such as total soluble solids, total acidity and pH during ripening and at harvest occurred among treatments. Many other studies confirm that organic and biodynamic viticulture, respectively, have little influence on grape composition [10,52,54,56,58,61]. Organically grown tomatoes [25,26] or other organically grown fruits such as strawberries [18] or apples [17], in contrast, showed higher quality. This might be highly dependent on the culture, management and physiological response of the plant. Leaf-area-to-fruit-weight-ratio in 2012 did not differ significantly among treatments in this study. Leaf-area-to-fruit-weight-ratios calculated in this study are high in comparison to values from other cultivars under semi-arid conditions [85,110], but varieties such as Gewürztraminer under cool climate conditions showed a high leaf-area-to-fruit-weight-ratio, too [111]. The fact that no differences in leaf-area-to-fruit-weight-ratios among treatments were observed might be one reason why treatments did not differ significantly in major berry quality traits. Another reason for this might be that physiological performance after veraison which influences the maturity of the fruit [107] did not differ highly among treatments (Fig 2). Nonetheless other berry quality parameters such as phenol content or aroma components might differ among the viticultural management systems because of differences in vigor. It should be further investigated as to whether grapes of different management systems differ in berry quality parameters highly linked to light interception by the canopy and translucency of the bunch zone [112].

Disease frequency of Botrytis was significantly increased in the biodynamic treatment compared to the integrated treatment where botryticides were applied. The differences in the management between the integrated and the biodynamic treatment include soil management, cover crop, plant protection strategy and the application of the biodynamic preparations. This means that the application of the preparations cannot entirely account for the observed differences, since the organic and the biodynamic treatments did not differ significantly in disease frequency of Botrytis in this trial. Moreover, the differences in plant protection strategy, e.g. the application of botryticides in the integrated management system, cannot entirely account for the observed differences since the integrated and the organic plot do not differ significantly either. Danner [56], in contrast, reports a higher disease frequency of Botrytis for organic management compared to conventional and biodynamic management from 1979–1983 in Austria (*Vitis vinifera* L cv. Grüner Veltliner).

Once Botrytis attacks the berries there is the risk of further fungi or bacteria entering the cracked barrier of the berry skin. One of the most frequent pathogens that severely endanger fruit and wine quality are acetic acid bacteria which cause sour rot [112,113]. Disease frequency of sour rot was significantly increased in the integrated treatment in 2010 and 2011, where sour rot on bunches occurred. One reason for this might be that copper, which was applied as a plant protection agent in the organic and the biodynamic plots until veraison (S5 Table), has a negative impact on growth of acetic acid bacteria which cause sour rot. In 2011 the monitoring results were confirmed by the gravimetrical determination of the amount of berries per vine affected by sour rot. The integrated treatment showed a significantly higher amount of infected yield (data not shown). Still further research is needed to verify whether copper may account for the observed differences concerning sour rot.

The biodynamic treatment showed a significantly higher content of primary amino acids in healthy berries during maturation compared to the integrated treatment. At harvest in 2010 all treatments showed sufficiently high concentrations of primary amino acids over 140 mg N L^-1^ to support completion of fermentation [88]. In 2011 contents of primary amino acids were generally low for all treatments. The organic and the biodynamic treatments showed a higher content of primary amino acids in 2012 compared to the integrated treatment. This may be partially due to the high yield loss in the organic and the biodynamic treatments in 2012, which was highest in the seasons of interest (Table 3). One reason for the lowest concentration of N-OPA in healthy berries of the integrated treatment might be the application of systemic fungicides. Oliva et al. [114] showed that the application of certain systemic fungicides significantly reduces total amino acid content as well as up to 11 out of 16 analyzed amino acids in grapes (*Vitis vinifera* L. cv. Monastrell). Especially fungicides that contained famoxadone or fenhexamid decreased the amino acid concentration in grapes. Teldor which contains fenhexamid as an active agent against Botrytis was applied once in 2010 and 2012, respectively, and twice in 2011 in the integrated treatment. The concentrations that were applied were slightly dependent on the phenological stages of the vines (S4 Table), but corresponded to the ones of the study by Oliva et al. [114]. A decrease in amino acid concentration in the juice might not only have implications on the success of alcoholic fermentation, but may also affect wine aroma and other beneficial effects such as protein synthesis [115]. However, the fungicide application alone cannot account for the observed differences in N-OPA, because the organic treatment did not differ significantly in N-OPA from the integrated treatment. The amount of healthy berries might as well have influenced N-OPA since the integrated and the biodynamic treatment also differed in disease frequency of Botrytis. On one hand the application of botryticides in the integrated treatment lowered the infestation with Botrytis, but might on the other hand be partially responsible for the decline in amino acid content in berries during maturation. An interaction between the effect of the fungicide and the amount of healthy berries might have caused the observed differences in N-OPA. Another factor that is likely to have influenced the amount of Botrytis and the amount of available α-amino acids in the berries of the different treatments is the nitrogen content in the soil that was reflected in the nitrogen content in the leaf tissue. It should be further investigated if enzymes that share in conversion of nitrogen in the plant such as nitrate reductase show different activities in vines of the different treatments.

## Conclusions

Growth and yield of grapevines under organic and biodynamic management decreased in comparison to the integrated treatment in this study, whereas fruit quality was not affected by the management system. Use of biodynamic preparations had little effects on vine growth and yield.

Since physiological performance was significantly higher under integrated management, it can be deduced that it influenced both growth, cluster weight, and berry weight and therefore yield levels. Soil management and fertilization strategy are likely to regulate physiological performance of the vines. Whether the changes in physiological performance occur due to hydraulic or chemical signals, such as phytohormones, should be further investigated. The discovery of reduced physiological performance of organically and biodynamically grown grapevines under field-conditions might potentially provide hints for further research on physiological performance of other organically grown perennial crops to better understand and further develop organic management strategies. Since a reduction of physiological performance in the organic and the biodynamic treatments occurred most evidently after full-bloom, organic and biodynamic growers should minimize water consumption of the cover crop in this period through mulching or rolling, because in this period berry size is determined and limited water availability might cause a reduction in cluster weight of the current and the subsequent year.

Nitrogen levels in the soil and in leaf tissues were also affected by the management system, but since the organic and the biodynamic treatments showed higher nitrogen levels, this factor cannot account for the observed reduction in growth and yield of the respective treatments. Nitrogen supply in the organic and the biodynamic treatments has been successfully ensured through cover crop management and compost addition.

Plant health differed significantly among treatments in this study due to the different plant protection strategies of the treatments investigated. In two out of three growing seasons disease incidence and severity of downy mildew in the organic and the biodynamic treatments partially accounted for yield reduction in the respective treatments. A stringent organic plant protection strategy with narrow intervals of spraying events especially in wet periods throughout the growing season is crucial to guarantee yield and fruit quality of grapevines.

Plant protection strategy also influenced nutrient status of the vines. Magnesium content in leaf tissues at veraison was significantly higher in the integrated treatment most likely due to the application of bitter salts. To which extend the higher magnesium content in the integrated treatment at veraison influenced physiological performance is subject of further research. Nonetheless, organic and biodynamic winegrowers should ensure sufficient magnesium supply to potentially enhance chlorophyll content and physiological performance of grapevines.

Since a growth reduction under organic and biodynamic management was observed in this study, further research on the microclimate in the bunch zone and secondary metabolites in berries related to radiation interception and translucency of the bunch zone should be conducted. Furthermore, sensory characteristics of the wines from the differing management systems should be compared.

## Supporting Information

S1 Dataset The underlying dataset of the trial. (ZIP)

S1 Fig Data of weather conditions during the seasons (A) 2010, (B) 2011, and (C) 2012. Daily average temperature [°C] and daily rainfall [mm]. Arrows indicate budbreak, full-bloom, veraison and harvest, respectively.
(TIF)

S2 Fig Maturity sampling during the seasons (A) 2010, (B) 2011, and (C) 2012. Total soluble solids [°Brix], total acidity [g L^-1^], and pH. Means ± sd.
(TIF)

S1 Table Results of the balanced fixed factorial analysis of variance (ANOVA with factors treatment and block) for the analysis of the soil samples in 2010 before data collection started [116]. *, ** and *** indicate statistical significance (p<0.05, p<0.01 and p<0.001) of the main effects determined by ANOVA (ns = not significant). Means ± sd per management system (int = integrated treatment, org = organic treatment, biodyn = biodynamic treatment).
(DOC)

S2 Table Analysis of residues of systemic plant protection agents on bunches in 2009. int = integrated treatment, org = organic treatment
(DOC)

S3 Table Components of the Wolff-mixture used as cover crop in the organic and the biodynamic treatment. (DOC)

S4 Table Pest and disease management of the integrated treatment. (DOC)

S5 Table Pest and disease management of the organic and the biodynamic treatment. (DOC)
