# Supplementary material for: Growth, Yield and Fruit Quality of Grapevines under Organic and Biodynamic Management
Source: PLoS One. 2015 Oct 8;10(10):e0138445. doi: 10.1371/journal.pone.0138445 (PMC4598136; doi:10.1371/journal.pone.0138445)
Supplement: S1 Table — *, ** and *** indicate statistical significance (p<0.05, p<0.01 and p<0.001) of the main effects determined by ANOVA (ns = not significant). Means ± sd per management system (int = integrated treatment, org = organic treatment, biodyn = biodynamic treatment). (DOC) [file pone.0138445.s004.doc]

**Supporting Information**

**S1 Table: Results of the balanced fixed factorial analysis of variance (ANOVA with factors treatment and block) for the analysis of the soil samples in 2010 before data collection started [116]**.

| parameter | sampling depth [cm] | unit | treatment | int | org | biodyn | block |
| --- | --- | --- | --- | --- | --- | --- | --- |
| soil moisture | 0-30 | [m-%] | ns | 16.6 ± 0.97 | 16.39 ± 0.81 | 16.12 ± 1.34 | ns |
|  | 30-60 | [m-%] | ns | 16.26 ± 1.06 | 16.13 ± 1.52 | 17.63 ± 1.92 | ns |
| pH | 0-30 |  | ns | 7.13 ± 0.32 | 7.1 ± 0.18 | 7.23 ± 0.1 | ns |
|  | 30-60 |  | ns | 7.3 ± 0.26 | 7.3 ± 0.22 | 7.38 ± 0.05 | ns |
| humus content | 0-30 | [m-%] | ns | 2.03 ± 0.65 | 2.58 ± 0.56 | 2.4 ± 0.55 | ns |
| C/N-ratio | 0-30 |  | ns | 18 ± 2.58 | 23.5 ± 10.21 | 19.5 ± 3.32 | ns |
|  | 30-60 |  | ns | 23.38 ± 9.45 | 18.13 ± 3.92 | 22 ± 4.97 | ns |
| phosphorus content | 0-30 | [mg P_2_O_5_/100g soil] | ns | 75.75 ± 4.11 | 80.75 ± 10.4 | 69.5 ± 13.82 | ns |
|  | 30-60 | [mg P_2_O_5_/100g soil] | ns | 83.5 ± 13.7 | 85.75 ± 9.91 | 89 ± 9.83 | ns |
| magnesium content | 0-30 | [mg Mg/100g soil] | ns | 12.5 ± 2.52 | 12.25 ± 0.5 | 11.75 ± 1.5 | ns |
|  | 30-60 | [mg Mg/100g soil] | ns | 14.25 ± 2.5 | 13.5 ± 2.08 | 13.5 ± 1 | ns |
| potassium content | 0-30 | [mg K/100g soil] | ns | 43.75 ± 10.78 | 46.75 ± 2.5 | 46.5 ± 5.26 | ns |
|  | 30-60 | [mg K/100g soil] | ns | 36.75 ± 5.44 | 40 ± 2 | 40.75 ± 4.19 | ns |

*, ** and *** indicate statistical significance (p<0.05, p<0.01 and p<0.001) of the main effects determined by ANOVA (ns = not significant). Means ± sd per management system (int=integrated treatment, org=organic treatment, biodyn=biodynamic treatment).

S2: Analysis of residues of systemic plant protection agents on bunches in 2009.

|  |  |  |  |  |  |
| --- | --- | --- | --- | --- | --- |
|  |  |  |  |  |  |
|  |  |  |  |  |
|  |  |  |  |  |  |
|  |  |  |  |  |
|  |  |  |  |  |
|  |  |  |  |  |
|  |  |  |  |  |  |
|  |  |  |  |  |
|  |  |  |  |  |
|  |  |  |  |  |

S3: Components of the Wolff-mixture used as cover crop in the organic and the biodynamic treatment.

|  |  |
| --- | --- |
|  |  |
|  |  |
|  |  |
|  |  |
|  |  |
|  |  |
|  |  |
|  |  |
|  |  |
|  |  |
|  |  |
|  |  |
|  |  |
|  |  |
|  |  |
|  |  |
|  |  |
|  |  |
|  |  |
|  |  |
|  |  |
|  |  |
|  |  |
|  |  |
|  |  |
|  |  |
|  |  |
|  |  |
|  |  |
|  |  |

|  |  |  |  |  |  |
| --- | --- | --- | --- | --- | --- |
|  |  |  |  |  |  |
|  |  |  |  |  |
|  |  |  |  |  |
|  |  |  |  |  |
|  |  |  |  |  |
|  |  |  |  |  |
|  |  |  |  |  |
|  |  |  |  |  |
|  |  |  |  |  |
|  |  |  |  |  |
|  |  |  |  |  |
|  |  |  |  |  |
|  |  |  |  |  |
|  |  |  |  |  |
|  |  |  |  |  |
|  |  |  |  |  |
|  |  |  |  |  |
|  |  |  |  |  |
|  |  |  |  |  |
|  |  |  |  |  |
|  |  |  |  |  |
|  |  |  |  |  |  |
|  |  |  |  |  |
|  |  |  |  |  |
|  |  |  |  |  |
|  |  |  |  |  |
|  |  |  |  |  |
|  |  |  |  |  |
|  |  |  |  |  |
|  |  |  |  |  |
|  |  |  |  |  |
|  |  |  |  |  |
|  |  |  |  |  |
|  |  |  |  |  |
|  |  |  |  |  |
|  |  |  |  |  |
|  |  |  |  |  |
|  |  |  |  |  |
|  |  |  |  |  |
|  |  |  |  |  |
|  |  |  |  |  |
|  |  |  |  |  |
|  |  |  |  |  |
|  |  |  |  |  |
|  |  |  |  |  |  |
|  |  |  |  |  |
|  |  |  |  |  |
|  |  |  |  |  |
|  |  |  |  |  |
|  |  |  |  |  |
|  |  |  |  |  |
|  |  |  |  |  |
|  |  |  |  |  |
|  |  |  |  |  |
|  |  |  |  |  |
|  |  |  |  |  |
|  |  |  |  |  |
|  |  |  |  |  |
|  |  |  |  |  |
|  |  |  |  |  |
|  |  |  |  |  |
|  |  |  |  |  |
|  |  |  |  |  |
|  |  |  |  |  |
|  |  |  |  |  |

|  |  |  |  |  |  |  |
| --- | --- | --- | --- | --- | --- | --- |
|  |  |  |  |  |  |  |
|  |  |  |  |  |  |
|  |  |  |  |  |  |
|  |  |  |  |  |  |
|  |  |  |  |  |  |
|  |  |  |  |  |  |
|  |  |  |  |  |  |
|  |  |  |  |  |  |
|  |  |  |  |  |  |
|  |  |  |  |  |  |
|  |  |  |  |  |  |
|  |  |  |  |  |  |
|  |  |  |  |  |  |
|  |  |  |  |  |  |
|  |  |  |  |  |  |
|  |  |  |  |  |  |
|  |  |  |  |  |  |
|  |  |  |  |  |  |
|  |  |  |  |  |  |
|  |  |  |  |  |  |
|  |  |  |  |  |  |
|  |  |  |  |  |  |
|  |  |  |  |  |  |
|  |  |  |  |  |  |
|  |  |  |  |  |  |
|  |  |  |  |  |  |
|  |  |  |  |  |  |
|  |  |  |  |  |  |
|  |  |  |  |  |  |
|  |  |  |  |  |  |
|  |  |  |  |  |  |
|  |  |  |  |  |  |
|  |  |  |  |  |  |
|  |  |  |  |  |  |  |
|  |  |  |  |  |  |
|  |  |  |  |  |  |
|  |  |  |  |  |  |
|  |  |  |  |  |  |
|  |  |  |  |  |  |
|  |  |  |  |  |  |
|  |  |  |  |  |  |
|  |  |  |  |  |  |
|  |  |  |  |  |  |
|  |  |  |  |  |  |
|  |  |  |  |  |  |
|  |  |  |  |  |  |
|  |  |  |  |  |  |
|  |  |  |  |  |  |
|  |  |  |  |  |  |
|  |  |  |  |  |  |
|  |  |  |  |  |  |
|  |  |  |  |  |  |
|  |  |  |  |  |  |
|  |  |  |  |  |  |
|  |  |  |  |  |  |
|  |  |  |  |  |  |
|  |  |  |  |  |  |
|  |  |  |  |  |  |
|  |  |  |  |  |  |
|  |  |  |  |  |  |
|  |  |  |  |  |  |
|  |  |  |  |  |  |
|  |  |  |  |  |  |
|  |  |  |  |  |  |  |
|  |  |  |  |  |  |
|  |  |  |  |  |  |
|  |  |  |  |  |  |
|  |  |  |  |  |  |
|  |  |  |  |  |  |
|  |  |  |  |  |  |
|  |  |  |  |  |  |
|  |  |  |  |  |  |
|  |  |  |  |  |  |
|  |  |  |  |  |  |
|  |  |  |  |  |  |
|  |  |  |  |  |  |
|  |  |  |  |  |  |
|  |  |  |  |  |  |
|  |  |  |  |  |  |
|  |  |  |  |  |  |
|  |  |  |  |  |  |
|  |  |  |  |  |  |
|  |  |  |  |  |  |
|  |  |  |  |  |  |
|  |  |  |  |  |  |
|  |  |  |  |  |  |
