# Supplementary material for: Growth, Yield and Fruit Quality of Grapevines under Organic and Biodynamic Management
Source: PLoS One. 2015 Oct 8;10(10):e0138445. doi: 10.1371/journal.pone.0138445 (PMC4598136; doi:10.1371/journal.pone.0138445)
Supplement: S2 Table — int = integrated treatment, org = organic treatment (DOC) [file pone.0138445.s005.doc]

**Supporting Information**

**S2 Table**: Analysis of residues of systemic plant protection agents on bunches in 2009.

| implementation against | active agent | maximum residue level [mg kg^-1^ fresh weight] | lower detection limit 2008 [mg kg^-1^] | int [mg kg^-1^] | org [mg kg^-1^] |
| --- | --- | --- | --- | --- | --- |
| *Erysiphe necator* | Spiroxamin | 1 | 0.01 | 0.029 | ns |
|  | Myclobutanil | 1 | 0.01 | 0.063 | ns |
| *Plasmopara viticola* | Famoxadon | 2 | 0.01 | 0.017 | ns |
|  | Dimethomorph | 3 | 0.01 | ns | ns |
|  | Folpet | 5 | 0.13 | 0.012 | ns |
|  | Dithiocarbamat | 5 | 0.01 | ns | ns |
| *Botrytis cinerea* | Fludioxonil | 2 | 0.01 | ns | ns |
|  | Pyrimethanil | 5 | 0.01 | 0.087 | ns |
|  | Cyprodinil | 5 | 0.01 | ns | ns |
|  | Fenhexamid | 5 | 0.01 | 0.53 | ns |

int=integrated treatment, org=organic treatment
