# Supplementary material for: Growth, Yield and Fruit Quality of Grapevines under Organic and Biodynamic Management
Source: PLoS One. 2015 Oct 8;10(10):e0138445. doi: 10.1371/journal.pone.0138445 (PMC4598136; doi:10.1371/journal.pone.0138445)
Supplement: S3 Table — (DOC) [file pone.0138445.s006.doc]

**Supporting Information**

**S3 Table**: Components of the Wolff-mixture used as cover crop in the organic and the biodynamic treatment.

| **main components** | **consistent of** |
| --- | --- |
| 7,5 % *Trifolium alexandrinum* |  |
| 7,5 % *Trifolium incarnatum* |  |
| 2,5 % *Phacelia tanacetifolia* |  |
| 20 % *Lathyrus latifolius* |  |
| 7,5 % *Melilotus albus* |  |
| 15 % *Onobrychis spec.* |  |
| 7,5 % *Medicago sativa* |  |
| 5 % *Medicago lupulina* |  |
| 5 % *Trifolium resupinatum* |  |
| 2,5 % *Trifolium hybridum* |  |
| 10 % "Bienenweidenmischung": | *Phacelia spec.* |
|  | *Fagopyrum esculentum* |
|  | *Coriandrum sativum* |
|  | *Calendula officinalis* |
|  | *Nigella sativa* |
|  | *Raphanus sativus var. Oleiformis Pers.* |
|  | *Malva sylvestris* |
|  | *Borago officinalis* |
|  | *Anethum graveolens* |
|  | *Helianthus annuus* |
| 10 % "Würzmischung": | *Sanguisorba minor* |
|  | *Carum carvi* |
|  | *Plantago lanceolata* |
|  | *Cichorium intybus* |
|  | *Achillea millefolium* |
|  | *Daucus carota subsp. carota* |
|  | *Petroselinum crispum* |
|  | *Foeniculum vulgare* |
|  | *Pastinaca sativa* |
|  | *Lotus corniculatus* |
