# Supplementary material for: Growth, Yield and Fruit Quality of Grapevines under Organic and Biodynamic Management
Source: PLoS One. 2015 Oct 8;10(10):e0138445. doi: 10.1371/journal.pone.0138445 (PMC4598136; doi:10.1371/journal.pone.0138445)
Supplement: S4 Table — (DOC) [file pone.0138445.s007.doc]

**Supporting Information**

**S4 Table: Pest and disease management of the integrated treatment.**

| year | date | agent | quantity of agent | unit | amount of water [L ha^-1^] |
| --- | --- | --- | --- | --- | --- |
| 2012 | 04.16.12 | RAK® 1+2 M | 500 | dispensers ha^-1^ | - |
|  | 05.21.12 | Delan WG | 0.3 | kg ha^-1^ | 150 |
|  |  | wettable sulfur | 3.6 | kg ha^-1^ |  |
|  | 06.04.12 | Cabrio Top | 1.6 | kg ha^-1^ | 225 |
|  | 06.18.12 | Forum Gold | 1.2 | kg ha^-1^ | 400 |
|  |  | Luna Expirence | 0.313 | L ha^-1^ |  |
|  | 06.29.12 | Profiler | 2.3 | kg ha^-1^ | 500 |
|  |  | Collis | 0.48 | L ha^-1^ |  |
|  | 07.05.12 | Calcium nitrate | 146 | kg ha^-1^ |  |
|  | 07.12.12 | Teldor (in bunch zone) | 0.6 | kg ha^-1^ | 200 |
|  | 07.10.12 | Profiler | 2.3 | kg ha^-1^ | 500 |
|  |  | Prosper | 0.7 | L ha^-1^ |  |
|  | 07.20.12 | Forum Star | 2.3 | kg ha^-1^ | 500 |
|  |  | Luna Expirence | 0.44 | L ha^-1^ |  |
|  | 08.02.12 | Mildicut | 4 | L ha^-1^ | 500 |
|  |  | Systhane | 0.24 | L ha^-1^ |  |
|  |  | Magnisal | 4 | kg ha^-1^ |  |
|  | 08.14.12 | Mildicut | 4 | L ha^-1^ | 500 |
|  |  | Systhane | 0.24 | L ha^-1^ |  |
|  |  | Magnisal | 4 | kg ha^-1^ |  |
|  | 08.21.12 | Cantus (in bunch zone) | 0.5 | kg ha^-1^ |  |
| 2011 | 04.18.11 | RAK® 1+2 M | 500 | dispensers ha^-1^ | - |
|  | 05.11.11 | wettable sulfur | 3.6 | kg ha^-1^ | 210 |
|  |  | Polyram WG | 1.2 | kg ha^-1^ |  |
|  | 05.25.11 | Folpan | 0.8 | kg ha^-1^ | 210 |
|  |  | Vivando | 0.16 | L ha^-1^ |  |
|  | 06.06.11 | Folpan 80 WDG 0,1 | 1 | kg ha^-1^ | 400 |
|  |  | Prosper | 0.5 | L ha^-1^ |  |
|  |  | wettable sulfur | 2 | kg ha^-1^ |  |
|  |  | Teldor | 1 | kg ha^-1^ |  |
|  | 06.15.11 | Cabrio Top | 2.4 | kg ha^-1^ | 400 |
|  |  | urea | 4 | kg ha^-1^ |  |
|  | 06.24.11 | Switch | 0.72 | kg ha^-1^ | 200 |
|  | 06.28.11 | Forum Gold | 1.56 | kg ha^-1^ | 400 |
|  |  | Vento Power | 1.4 | L ha^-1^ |  |
|  |  | urea | 4 | kg ha^-1^ |  |
|  | 07.11.11 | Mildicut | 4 | L ha^-1^ | 500 |
|  |  | Topas | 0.32 | L ha^-1^ |  |
|  |  | urea | 5 | kg ha^-1^ |  |
|  |  | bitter salts | 10 | kg ha^-1^ |  |
|  | 07.26.11 | Mildicut | 4 | L ha^-1^ | 500 |
|  |  | Topas | 0.32 | L ha^-1^ |  |
|  |  | bitter salts | 10 | kg ha^-1^ |  |
|  | 08.11.11 | Teldor | 0.64 | kg ha^-1^ | 200 |
| 2010 | 04.19.10 | RAK® 1+2 M | 500 | dispensers ha^-1^ | - |
|  | 05.25.10 | wettable sulfur | 3.6 | kg ha^-1^ | 155 |
|  |  | Polyram WG | 1.2 | L ha^-1^ |  |
|  | 06.08.10 | wettable sulfur | 3.6 | kg ha^-1^ | 210 |
|  |  | Vivando | 0.12 | L ha^-1^ |  |
|  |  | Forum Gold | 0.72 | kg ha^-1^ |  |
|  | 06.21.10 | wettable sulfur | 2 | kg ha^-1^ | 400 |
|  |  | Cabrio Top | 2 | kg ha^-1^ |  |
|  | 06.26.10 | Calcium nitrate | 292 | kg ha^-1^ |  |
|  | 06.29.10 | Forum Gold | 1.44 | kg ha^-1^ | 500 |
|  |  | Vivando | 0.24 | L ha^-1^ |  |
|  | 07.08.10 | Scala | 1.75 | kg ha^-1^ | 500 |
|  |  | Cabrio Top | 2.8 | kg ha^-1^ |  |
|  | 07.20.10 | Fantic F | 2.4 | kg ha^-1^ | 500 |
|  |  | Talendo | 0.4 | L ha^-1^ |  |
|  | 08.02.10 | Systhane 20 EW | 0.24 | L ha^-1^ | 500 |
|  |  | Mildicut | 4 | L ha^-1^ |  |
|  | 08.13.10 | bitter salts (Mg) | 10 | kg ha^-1^ |  |
|  | 08.18.10 | Systhane 20 EW | 0.24 | L ha^-1^ | 500 |
|  |  | Mildicut | 4 | L ha^-1^ |  |
|  |  | Teldor | 1.6 | kg ha^-1^ |  |
