# Supplementary material for: Growth, Yield and Fruit Quality of Grapevines under Organic and Biodynamic Management
Source: PLoS One. 2015 Oct 8;10(10):e0138445. doi: 10.1371/journal.pone.0138445 (PMC4598136; doi:10.1371/journal.pone.0138445)
Supplement: S5 Table — (DOC) [file pone.0138445.s008.doc]

**Supporting Information**

**S5 Table: Pest and disease management of the organic and the biodynamic treatment.**

| year | date | agent | amount of copper [g ha^-1^] | quantity of agent | unit | amount of water [L ha^-1^] |
| --- | --- | --- | --- | --- | --- | --- |
| 2012 | 04.16.12 | RAK® 1+2 M |  | 500 | dispensers ha^-1^ | - |
|  | 05.18.12 | wettable sulfur |  | 2.4 | kg ha^-1^ | 155 |
|  |  | Mycosin VIN |  | 2 | kg ha^-1^ |  |
|  | 05.29.12 | wettable sulfur |  | 3.6 | kg ha^-1^ | 230 |
|  |  | Mycosin VIN |  | 3.5 | kg ha^-1^ |  |
|  | 05.06.12 | wettable sulfur |  | 4.8 | kg ha^-1^ | 230 |
|  |  | Funguran | 300 |  |  |  |
|  | 06.12.12 | wettable sulfur |  | 2.4 | kg ha^-1^ | 300 |
|  |  | Funguran | 300 |  |  |  |
|  | 06.19.12 | wettable sulfur |  | 3.6 | kg ha^-1^ | 400 |
|  |  | Cuprozin | 300 | 1 | kg ha^-1^ |  |
|  | 06.27.12 | wettable sulfur |  | 2.4 | kg ha^-1^ | 500 |
|  |  | Vitisan/Saukarb |  | 6 | kg ha^-1^ |  |
|  |  | Cuprozin | 500 | 1.67 | kg ha^-1^ |  |
|  | 07.06.12 | AlgoVital |  | 1.5 | L ha^-1^ | 500 |
|  |  | Funguran | 500 | 1.1 | kg ha^-1^ |  |
|  |  | wettable sulfur |  | 3.2 | kg ha^-1^ |  |
|  |  | water glass |  | 3 | L ha^-1^ |  |
|  | 07.16.12 | AlgoVital |  | 1.5 | L ha^-1^ | 500 |
|  |  | Funguran | 500 | 1.1 | kg ha^-1^ |  |
|  |  | wettable sulfur |  | 3.2 | kg ha^-1^ |  |
|  |  | water glass |  | 3 | L ha^-1^ |  |
|  | 07.23.12 | wettable sulfur |  | 3.2 | kg ha^-1^ | 500 |
|  |  | Funguran | 500 | 1.1 | kg ha^-1^ |  |
|  |  | Salukarb/Vitisan |  | 6 | kg ha^-1^ |  |
|  |  | AlgoVital |  | 1.5 | L ha^-1^ |  |
|  | 08.02.12 | wettable sulfur |  | 3.2 | kg ha^-1^ | 500 |
|  |  | Funguran Progress | 150 | 0.425 | kg ha^-1^ |  |
|  |  | Salukarb/Vitisan |  | 6 | kg ha^-1^ |  |
|  |  | AlgoVital |  | 1.5 | L ha^-1^ |  |
|  | 08.13.12 | Funguran Progress | 150 | 0.425 | kg ha^-1^ | 500 |
|  |  | Salukarb/Vitisan |  | 8 | kg ha^-1^ |  |
|  |  | AlgoVital |  | 1.5 | L ha^-1^ |  |
| 2011 | 04.18.11 | RAK® 1+2 M |  | 500 | dispensers ha^-1^ | - |
|  | 05.12.11 | wettable sulfur |  | 3.6 | kg ha^-1^ | 230 |
|  |  | Mycosin VIN |  | 3 | kg ha^-1^ |  |
|  | 04.23.06 | wettable sulfur |  | 3.6 | kg ha^-1^ | 230 |
|  |  | Mycosin VIN |  | 3 | kg ha^-1^ |  |
|  | 06.03.11 | wettable sulfur |  | 3.6 | kg ha^-1^ | 400 |
|  |  | Mycosin VIN |  | 3 | kg ha^-1^ |  |
|  | 06.10.11 | wettable sulfur |  | 2.4 | kg ha^-1^ | 400 |
|  |  | Funguran | 300 | 0.66 | kg ha^-1^ |  |
|  |  | Salukarb |  | 6 | kg ha^-1^ |  |
|  | 06.17.11 | wettable sulfur |  | 2.8 | kg ha^-1^ | 400 |
|  |  | Funguran | 500 | 1.1 | kg ha^-1^ |  |
|  |  | Salukarb |  | 6 | kg ha^-1^ |  |
|  | 06.27.11 | wettable sulfur |  | 3.2 | kg ha^-1^ | 500 |
|  |  | Cuprozin | 500 | 1.66 | L ha^-1^ |  |
|  |  | Salukarb |  | 6 | kg ha^-1^ |  |
|  | 07.05.11 | wettable sulfur |  | 3.2 | kg ha^-1^ | 500 |
|  |  | Cuprozin | 300 | 1 | L ha^-1^ |  |
|  |  | Salukarb |  | 6 | kg ha^-1^ |  |
|  |  | ProFital fluid 0,15% |  | 0.75 | L ha^-1^ |  |
|  | 07.15.11 | wettable sulfur |  | 2 | kg ha^-1^ | 500 |
|  |  | Funguran | 300 | 0.66 | kg ha^-1^ |  |
|  |  | Salukarb |  | 8 | kg ha^-1^ |  |
|  |  | ProFital fluid 0,15% |  | 0.75 | L ha^-1^ |  |
|  | 07.25.11 | Funguran | 300 | 0.66 | kg ha^-1^ | 500 |
|  |  | Salukarb |  | 8 | kg ha^-1^ |  |
|  |  | ProFital fluid 0,15% |  | 0.75 | L ha^-1^ |  |
|  | 08.10.11 | Funguran | 300 | 0.66 | kg ha^-1^ | 500 |
|  |  | Salukarb |  | 8 | kg ha^-1^ |  |
|  |  | ProFital fluid 0,15% |  | 0.75 | L ha^-1^ |  |
| 2010 | 04.19.10 | RAK® 1+2 M |  | 500 | dispensers ha^-1^ | - |
|  | 05.25.10 | wettable sulfur |  | 3.6 | kg ha^-1^ | 155 |
|  |  | Mycosin VIN |  | 3 | kg ha^-1^ |  |
|  | 05.29.10 | Mycosin VIN |  | 3 | kg ha^-1^ | 155 |
|  | 06.04.10 | wettable sulfur |  | 3.6 | kg ha^-1^ | 155 |
|  |  | Mycosin VIN |  | 3 | kg ha^-1^ |  |
|  | 06.15.10 | wettable sulfur |  | 4.8 | kg ha^-1^ | 230 |
|  |  | Funguran | 250 | 0.56 | kg ha^-1^ |  |
|  | 06.22.10 | wettable sulfur |  | 4.8 | kg ha^-1^ | 400 |
|  |  | Funguran | 250 | 0.56 | kg ha^-1^ |  |
|  | 06.29.10 | wettable sulfur |  | 4 | kg ha^-1^ | 500 |
|  |  | Cuprozin | 450 | 1.5 | L ha^-1^ |  |
|  | 07.08.10 | wettable sulfur |  | 2.8 | kg ha^-1^ | 500 |
|  |  | Cuprozin | 450 | 1.5 | L ha^-1^ |  |
|  |  | Salukarb |  | 6 | kg ha^-1^ |  |
|  | 07.16.10 | wettable sulfur |  | 3.2 | kg ha^-1^ | 500 |
|  |  | Funguran | 400 | 0.88 | kg ha^-1^ |  |
|  |  | Salukarb |  | 6 | kg ha^-1^ |  |
|  | 07.26.10 | wettable sulfur |  | 3.2 | kg ha^-1^ | 500 |
|  |  | Funguran | 300 | 0.66 | kg ha^-1^ |  |
|  |  | Salkcarb |  | 6 | kg ha^-1^ |  |
|  | 08.06.10 | Funguran | 300 | 0.66 | kg ha^-1^ | 500 |
|  |  | Salukarb |  | 12 | kg ha^-1^ |  |
